# Challenges and Innovations in Phase I Dose-Finding Designs for Molecularly Targeted Agents and Cancer Immunotherapies

**DOI:** 10.4172/2155-6180.1000324

**Published:** 2016-11-14

**Authors:** Jun Yin, Shihao Shen

**Affiliations:** 1 Department of Health Sciences Research, Mayo Clinic, Rochester, MN 55905, USA; 2 Department of Microbiology, Immunology, & Molecular Genetics, UCLA, Los Angeles, CA 90024, USA

**Keywords:** Phase I, Dose-finding, Toxicity score, Late toxicity, Cumulative toxicity, Oncology, Molecularly targeted agent, Cancer immunotherapy

## Abstract

Phase I oncology trials are designed to identify a safe dose with an acceptable toxicity profile. In traditional phase I dose-finding design, the dose is typically determined based on the probability of severe toxicity observed during the first treatment cycle. The recent development of molecularly targeted agents and cancer immunotherapies call for new innovations in phase I designs, because of prolonged treatment cycles often involved. Various phase I designs using toxicity and efficacy endpoints from multiple treatment cycles have been developed for these new treatment agents. Here, we will review the novel endpoints and designs for the phase I oncology clinical trials.

## Introduction

Phase I clinical trials are designed to identify the recommended phase II dose (RP2D) for follow-up trials. Typically, the efficacy of the cytotoxic agents is positively correlated with the toxicity. Therefore, the recommended dose is generally the maximum-tolerated dose (MTD) for cytotoxic agents. Usually, the dose-limiting toxicity (DLT) events are grade 3 or 4 toxicity events in the first treatment cycles. The DLT-based toxicity endpoints are used to determine MTD. Toxicity events from late treatment cycles are often recorder but not used in the dose-finding.

The molecular mechanisms of cancer prognosis and immune checkpoints leads to the rapid development of molecular targeted agents (MTAs) and cancer immunotherapies that have the potentials to greatly improve the cancer treatment. It is estimated that more than 700 oncology drugs are currently under development, with the majority of them being MTAs and cancer immunotherapies. However, a very low percentage of the new drugs will eventually demonstrate sufficient efficacy and tolerable toxicity for regulatory approval of clinical application. The eventual approval rate for drugs tested in oncology phase I trials is less than 7%, being the lowest approval rates of all diseases [1]. In addition, due to the slow recruitment rates and long follow-up time, the oncology clinical trials are estimated to take averagely more than one year longer than other diseases. These challenges in the oncology clinical trials call for innovations in the designs of clinical trials in oncology to enhance the attrition rate of oncology drugs, especially the MTAs and the cancer immunotherapies under development.

The unique features of the MTAs and cancer immunotherapies require that novel toxicity endpoints to be considered in the phase I oncology clinical trials. Different from the cytotoxic agents that are often administrated in limited number of treatment cycles, MTA and immunotherapy administrations are often prolonged for many treatment cycles until disease progression [2]. The toxicity profile of MTAs and cancer immunotherapies are often significantly different from cytotoxic agents, characterizing by chronic, prolonged events or cumulative toxicity as opposed to the early onset adverse events associated with cytotoxicity. A recent study observed that in 36 clinical trials of MTAs, more than half of the 445 patients developed their worse grade toxicity after the first cycle, in which more than half of the grade 3 or 4 toxicity events occurred after cycle 1 [3]. Therefore, in phase I clinical trials of MTAs and cancer immunotherapies, it is meaningful to account for late toxicity events after the first treatment cycle.

Besides the immediate harmful impact of server toxicity events of grade 3 or 4, repeated and chronic occurrence of lower grade events can also significantly impair patient’s life quality [4]. The prolonged treatment of MTAs and cancer immunotherapies deem it necessary to consider the lesser toxicity events with repeated occurrence in multiple treatment cycles. Since toxicity data are high dimensional in nature, with various types, grades, attribution and times of occurrence, dose-finding designs in phase I clinical trials could benefit from a new toxicity endpoint paradigm. A variety of toxicity scores have been proposed to address the comprehensive toxicity profiles by combining multiple types of toxicity events with various severities.

Different from the cytotoxic agents, the highest tolerated dose of MTAs or cancer immunotherapies may not pose significantly improved efficacy and clinical benefit over the lower doses. A retrospective analysis of over 600 patients involving in MTAs or immunotherapies based clinical trials showed similar response rate and survival outcomes between patients receiving lower and medium dose of MTAs [5]. Similar dose-response relationship has also been observed in early phase studies conducted in the UK [6].

In this article, we will review the recent development in the novel designs of phase I clinical trials in oncology. The review is divided into three sections. The first section reviews the recent developments in toxicity scores that summarize multiple toxicity grades and toxicity types. The second section reviews the novel phase I designs accounting for repeated measurement of toxicity events over multiple treatment cycles. The third section reviews the combination of early-stage efficacy endpoints with toxicity events in phase I dose-finding.

## Toxicity Score for the Multiple Toxicity Events

The traditional definition of DLT based on the grade 3 or 4 toxicity events cannot catch the lower grade toxicity events that may have profound impact on patient’s life quality during prolonged treatment cycles. In some cases, the co-occurrence of multiple lower grade toxicity events may lead to the eventual dropout of patients. Therefore, a variety of toxicity scoring systems have been proposed to measure quantitatively and comprehensively the overall severity of multiple toxicities for a patient, using an equivalent toxicity score (ETS) [7] or including total toxicity burden (TTB) [8,9].

Suppose that there are *k*=1…*K* patients, let *T_i,k_* be the *i*th (*i*=1…*k_I_*) toxicity event of the *k*th patients. ETS converts each toxicity event *T_i,k_* into a numerical value of adjusted grade *G_i,k_* predefined according to the severity of the toxicity event. For the patient with only one toxicity event, the EST score will be the adjusted grade minus 1, Si,k=Gi,k − 1. For the patient with multiple toxicity events, the EST score will be a logistic function that accumulating the other types of toxicities:

(1) $$e_{i,k}=\exp(\alpha +\beta (\sum (w_{i}G_{i,k})/G_{\max,k}-1))S_{i,k}=G_{\max,k}-1+\frac{e}{1+e}$$

TTB is defined as the sum of the weights of all toxicities experienced by a patient, where weight reflects the relative clinical importance of each grade and type of toxicity [7]. Similar to the TTB, the total toxicity profile (TTP) was proposed by Ezzalfani et al. [10] as the Euclidean norm of the weights, which is the endpoint used in this design. Again, let *w_lh_* denote the elicited weight of toxicity type *l* (*l* ∈ {1, …, *L*}) occurring at grade *h* (*h* ∈ {0, …, 4}). Hence, the weight vector for toxicity l is *w_l_*_=_(*w_l_*_0_, …, *w_l_*_4_)T and the weight matrix is denoted as *W*=(*w_1_*, …, *w_L_*)*T*. For patient i, denote the maximum observed grade of toxicity type *l* as *G_il_*. Then the *TTP_i_* is defined as

(2) $${\mathrm{TTP}}_{i}=\sqrt{\sum_{l=1}^{L}\sum_{h=0}^{4}w_{jh}^{2}1(G_{il}=h)}$$

where 1(*G_il_*=*h*) is an indicator function which takes value 1 if the maximum observed grade is *h* for toxicity type *l*, and 0 otherwise.

The TTP is further normalized to nTTP, in order to constrain the continuous toxicity endpoint to be within 0 and 1,

(3) $${\mathrm{nTTP}}_{i}=\frac{{\mathrm{TTP}}_{i}}{v}$$

Where *v* is a normalization constant that is equal to or slightly larger than the maximum TTP that occurs with the most severe toxicity profile. Further details about the specification of the normalization constant v are provided in Section 3.1 and Ezzalfani et al. [8].

## Measure Toxicity over Prolonged Treatment Cycles

Recent observations indicate that the majority of the DLT occurred after the first treatment cycle in multiple phase I oncology clinical trials. This provides the rationale for a variety of dose-finding designs to take into account toxicities from subsequent cycles of treatment.

Cheung and Chappell used a time-to-event model (TITE-CRM) for late on-set toxicities in a sequential dose-finding design [11]. TITE-CRM incorporated the time-to-event data of the DLT events into the CRM model. Patients that are still under the late-cycle treatments are considered as the censored events. Therefore, TITE-CRM framework allows the usage of various treatment cycles and partial treatment data to be used in the dose-finding calculation. Since most MTAs and immunotherapies involve prolonged treatments, it is necessary to model the dose-finding using partial data that are collected from the patients that are still under late-cycle treatments.

Doussau et al. proposed a dose-finding design using a longitudinal graded toxicity in phase I oncology trials [6]. In this design, decision rules on dose escalation were based on evaluation of ordinal toxicity in the mixed-effect proportional odds model after a run-in stage of 15 patients. Instead of modeling the time-to-event data of DLT events as in TITE-CRM, this model accounts for the repeated measurement data of lower grade toxicity in the early treatment cycles. The toxicity grades were modeled as an ordinal data representing lesser to more severe toxicity grades. At the end of the trial, this approach identifies the probability of a DLT toxicity event and the trend in the of toxicity over multiple treatment cycles.

A third approach was developed by Fernandes et al. using Markov chains to model DLTs in multiple treatment cycles [10]. A Markov approach is used to assess the dose variations over treatment cycles and estimate the toxicity from each cycle. The Markov model of probability of toxicity on any cycle is formulated based on the current and previous doses given no toxicity in previous cycles. This approach is designed to allow patients have between-cycle dose-escalation or de-escalation during multiple treatment cycles. For patient k on the cycle c given that the patient has not experienced any DLTs before the cycle c, the conditional probability of toxicity is

$$p_{i,c}=1-\exp-\alpha (d_{i,c}-\rho \;\max_{c}\;d_{i,c})-\beta D_{i,c}d_{i,c}$$

In which, *d_i,c_* is the assigned dose for the patient c at the treatment cycle c and *Di,c*= Σ *j*=1c − 1*d_i,j_* is the sum of the dose selection before the treatment cycle. The parameters *α* and *β* are regression parameters.

All the approaches above are based on the DLT toxicity events. If any patients experienced dose-limiting toxicity in any treatment cycles, the patients are removed for the following-up cycles. To account for the minor toxicity events (¡ grade 3) over multiple treatment cycles, a Bayesian phase I design was developed [12] to incorporate the total toxicity profile (TTP), a quasi-continuous toxicity endpoint, in the dose estimation from toxicity data of multiple treatment cycles. The TTP score captures a comprehensive toxicity profile including multiple types and grades of toxicities occurring during the first treatment cycle. The repeated measures design (RMD) using longitudinal toxicity data from multiple cycles (assuming independence across cycles) improved the ability to identify the MTD compared to a design that utilized data from just one cycle. It uses simulation and time trend estimation in a real phase I study to demonstrate that the information about the time trend of the toxicity profile is important to guide the drug administration over time.

## Dual-Modeling of Toxicity and Efficacy Endpoints

Despite the success of targeted agents such as bevacizumab, imatinib, and trastuzumab that demonstrated significant clinical benefit in several cancers, many other targeted agents such as ISIS 3521, R115777 and ZD1839 failed in clinical trials. The failure of many promising targeted agents in late-phase clinical trials has prompted a reconsideration of the standard dose finding designs that searches for the maximum-tolerated dose (MTD). Since the dose-efficacy curves are usually unknown and in many cases lower dose of MTAs and immunotherapies demonstrated similar efficacy as higher dose [5], the standard strategy of searching for MTD needs to be modified for MTAs and immunotherapies. New phase I oncology clinical trial designs are proposed to use both the toxicity and early efficacy endpoints in the dose-finding. This section will review these designs with both the toxicity and early efficacy endpoints.

The standard phase I designs are extending in two directions to account for both toxicity and efficacy endpoints in a phase I setting. The first approach models the clinical outcomes in a phase I study in a sequential order: 1. neither DLT nor efficacy events, 2. no DLT but with efficacy, 3. with DLT. The target of these phase I designs are to find the dose with the second type of events that demonstrated sufficient efficacy without DLT. These statistical models often use an ordinal trinary variable to describe these events.

Ivanova explored the trivariate clinical trial designs for the three ordinal outcomes of no toxicity and efficacy [13], no toxicity and efficacy and with toxicity. Their new design follows the framework of a play-the-winner design [14]. It repeats the dose if there is no toxicity and indication for efficacy. On the other side, the dose will be increased if no toxicity and efficacy is observed; and the dose will be decreased if toxicity is observed. The dose assignment is based on each subject. Suppose that the most recent subject *k* is assigned to dose level *d_k_*. The next subject *k* + 1 will be assigned to:

- dose *d_j_*−1 if the subject *k* experiences toxicity,
- dose *d_j_* if the subject *k* has early efficacy response and no toxicity,
- dose *d_j_*+1 if the subject *k* has no efficacy response.

Besides simple dose-escalation rule based on toxicity and efficacy endpoints, more complex statistical designs have been proposed to for the dual toxicity and efficacy dose-finding. The majority of the designs depend on modeling trivariate endpoints in the CRM designs [15–17], in which patient outcomes are represented by a trinary ordinal variable for both efficacy and toxicity. All the three models from Mandrekar et al. [16], Zhang et al. [17] and Thall et al. [15] incorporates the proportional odds regression model from McCullagh’s [18], also known as the cumulative odds model [18], in the CRM model. In details, suppose that *ψ*={*ψ*_0_, *ψ*_1_, *ψ*_2_} represents the tri possibilites of no toxicity and response, no toxicity but response and toxicity, Mandrekar et al. [16] models the proportional odds model by:

(4) $$\log(\frac{\psi_{1}}{\psi_{0}})=\mu +\alpha +\beta_{1}x,\log(\psi_{2})=\mu +\beta_{2}x.$$

In which, x is an indicator of the dose levels and the rest parameters represent the toxicity and efficacy relationship. Alternatively, Zhang et al. [17] models the proportional odds by:

(5) $$\log(\frac{\psi_{1}}{\psi_{0}})=\mu +\alpha +\beta_{1}x,\log(\frac{\psi_{2}}{\psi_{0}+\psi_{1}})=\mu +\beta_{2}x.$$

And Thall et al. [15–21] models the proportional odds by:

(6) $$\log(\psi_{1}+\psi_{2})=\mu +\alpha +\beta x,\log(\psi_{2})=\mu +\beta x.$$

The second approach models the bivariate structure of toxicity-efficacy outcomes through a joint modeling. The toxicity and efficacy outcomes are combined into a joint likelihood model with the parameter to represent the within-subject correlation between the toxicity and efficacy outcomes. The bivariate CRM (bCRM) [19] represents such an approach that extends the CRM by a marginal logit distribution of dose-toxicity and a marginal logit distribution of dose-disease progression, along with a bivariate distribution that links toxicity and progression outcomes.

Thall and Cook model the efficacy toxicity trade-off function in a dose-finding algorithm [20]. Suppose that the marginal distribution of the probability of toxicity *ψ_T_* is defined by and the probability of efficacy is defined by *ψ_T_* = *μ_T_* + *β_T_x* and the probability of efficacy is defined by *ψ_E_* = *μ_E_* + *β_E_x* + *β_E_x*^2^, in which x is the dose level. The joint probability defines the efficacy-toxicity trade-off is defined by:

$$\psi_{E,T}={\left(\psi_{E}\right)}^{E}{\left(1-\psi_{E}\right)}^{1-E}{\left(\psi_{T}\right)}^{T}{\left(1-\psi_{T}\right)}^{1-T}+{\left(-1\right)}^{E+T}\psi_{E}(1-\psi_{E})\psi_{T}(1-\psi_{T})\frac{e^{c}-1}{e^{c}+1}$$

The joint toxicity-efficacy distribution follows the “Gumbel” or “Morgenster” distribution [21], in which the c parameter defines the toxicity and efficacy trade-off.

Yin et al. used a dose-finding scheme based on the odds ratio of toxicity and efficacy [22]. Based on the odds ratio contour of toxicity and efficacy, a Bayesian adaptive decision-making was developed in this model. In details, the joint distribution of toxicity and efficacy is based on odds ratio of $\frac{\psi_{T}(1-\psi_{T})}{\psi_{E}(1-\psi_{E})}$ Compared to the trade-off function based on subjective criteria, this method leads to a intuitive interpretation of the odds ratio.

Bekele and Shen utilized the bivariate probit models [23], in which a patient’s toxicity and efficacy outcome are correlated with each other. A continuous latent variable was introduced for the joint modeling of the continuous efficacy endpoint and the binary toxicity endpoint in a bivariate model, at each given dose level. The joint modeling is based on the dependence between the two outcomes. In details, a bivariate normal distribution is used as the joint model of toxicity and efficacy:

$$f(\psi_{E},\psi_{T})=\prod_{k=1}^{K}\left\{\int P(\psi_{E_{k}},Z_{k})dZ_{k}\right\}$$

In which, P is a bivariate normal distribution and Z is a continuous latent variable related to the binary toxicity endpoint.

## Discussion

MTAs and cancer immunotherapies demonstrate different characteristics in phase I oncology clinical trials as the traditional cytotoxic agents. Usually MTAs and cancer immunotherapies last for longer treatment cycles than the cytotoxic agents, so that it becomes more important to account for the late-cycle toxicity events and lasting effect of minor toxicity events. Furthermore, MTAs and cancer immunotherapies demonstrate different toxicity-efficacy curve as the traditional cytotoxic, as lower doses of MTAs and cancer immunotherapies show similar early-stage efficacy endpoints as higher doses. As a result, alternative toxicity endpoints and early-stage efficacy end-points were developed for the phase I oncology clinical trials of MTAs and cancer immunotherapies.

Here, we review the alternative toxicity endpoints and early-stage efficacy endpoints for the phase I oncology clinical trials. As approaches to summarize the toxicity events from multiple toxicity types and utilize the minor toxicity grades, we review the equivalent toxicity score (ETS) [4], total toxicity burden (TTB) [2–14], and total toxicity probability (TTP) that combine the information from all toxicity grades and multiple toxicity types [10]. We also review the phase I strategy using toxicity endpoints from multiple treatment cycles. The multi-cycle toxicity data were modeled as time-to-event data by Cheung and Chappell [11], as longitudinal data by Doussau et al. [24] and as the time series data by Fernandes et al. [10]. As shown by Yin et al. the TTP toxicity score for multiple toxicity types and minor grades can also be incorporated in the designs for multi-cycle toxicity [22]. Simulation studies and preliminary analyses in real phase I data demonstrate that the information about the time trend of the toxicity profile is important to guide the drug administration over time.

Another important aspect of the novel phase I designs to consider the dual toxicity-efficacy endpoints for MTAs and cancer immunotherapies, since these treatments often demonstrate similar efficacy at several dose levels [5]. We review two major branches of the dual toxicity and efficacy approaches that either uses a trinary ordinal variable modeling the three possible responses of no response, efficacy response only and toxicity response [13,14]; alternatively, the second approach models the bivariate structure of toxicity-efficacy outcomes through a joint modeling with correlation parameter between toxicity and efficacy [19–23].

## Conclusion

To align with the advancement in the statistical designs of phase I oncology trials, it is essential to involve clinical investigators as these new designs are developed. The majority of the novel designs require setting the tunning parameters according to the clinical applications. For example, in order to develop a sensible toxicity score for multiple toxicity types, the weight matrix must consider the relative clinical importance of multiple toxicity types in the context of the tumor types in study. Since the alternative toxicity endpoints and early-stage efficacy endpoints show great promise to improve the phase I oncology dose-finding, it is imperative to continue the efforts to translate the theoretical research into the clinical practice.
